# Editorial: Physiological, molecular and genetic perspectives of chilling tolerance in horticultural crops, volume II

**DOI:** 10.3389/fpls.2022.1048923

**Published:** 2022-10-13

**Authors:** Isabel Lara, Diane M. Beckles, María F. Drincovich, Shifeng Cao

**Affiliations:** ^1^ Postharvest Unit, AGROTÈCNIO Center, Universitat de Lleida, Lleida, Spain; ^2^ Department of Plant Sciences, University of California, Davis, Davis, CA, United States; ^3^ Centro de Estudios Fotosintéticos y Bioquímicos - Consejo Nacional de Investigaciones Científicas y Técnicas (CEFOBI-CONICET), Rosario National University, Rosario, Argentina; ^4^ College of Biological and Environmental Sciences, Zhejiang Wanli University, Ningbo, China

**Keywords:** chilling response, chilling stress, chilling tolerance, horticultural crop, metabolomics, transcriptomics

## Introduction

For crops of tropical and subtropical origin, exposure to low temperatures often causes chilling injury symptoms, which can range from altered appearance (such as surface pitting and discoloration) to severe physiological disorders and impaired metabolism. To cope with suboptimal temperatures in the surrounding environment, crops have evolved complex mechanisms, which entail stress signal perception and transduction, activation of stress-responsive genes, and the synthesis of stress-related molecules.

Plant breeding programs have been instrumental in obtaining chilling-tolerant cultivars in a number of horticultural crops. More recently, the incorporation of molecular and omics-based techniques into conventional breeding procedures has vastly improved breeding strategies by enhancing the efficacy of screening for chilling tolerance-associated traits. Moreover, these new tools will boost knowledge of chilling responses and tolerance mechanisms, and the discovery of related pathways and genes. As a part of the “Physiological, Molecular and Genetic Perspectives of Chilling Tolerance in Horticultural Crops” series (Lara et al., 2020), this Research Topic was launched with the aim of offering an overview of recent developments in this area.

The papers in this collection explored the mechanisms involved in chilling tolerance in a number of plant species, including commercially important fruit crops such as pepper, tomato, banana and peach/nectarine, a medicinal plant (*Tetrastigma hemsleyanum*) and *Arabidopsis*. In addition to helping to reveal the mechanisms underlying cold stress tolerance, these findings provide the basis for future breeding programs, and offer clues for the alleviation of stress symptoms.

## Responses to cold stress

A cold-sensitive (‘XianSheng’) and a cold-resistant (‘GanZi’) cultivar were used to help dissect the molecular responses to cold stress, which significantly affects field production of pepper (*Capsicum annuum*) seedlings (Zhang et al.). Transcriptomic analyses identified over 10,000 differentially expressed genes, most of which were involved in amino acid biosynthesis, plant hormone signal transduction, and the mitogen-activated protein kinase (MAPK) signaling pathway. Differential metabolite accumulation included increased contents of free polyamines, abscisic and jasmonic acids, raffinose and proline in response to cold exposure. Reactive oxygen species (ROS) signaling was also a key factor in the regulation of cold stress responses. Another study on pepper (Wang et al.) demonstrated the importance of the *CaALAD* gene for cold tolerance. *CaALAD* encodes an aminolevulinate dehydratase, an essential protein for chlorophyll biosynthesis. Transgenic *Arabidopsis* plants overexpressing *CaALAD* displayed significantly higher transcription of elements in the ICE1-CBF-COR transcription network, a key pathway in plant response to cold (Hwarari et al., 2022), in parallel with decreased ROS contents and enhanced chlorophyll biosynthesis. Accordingly, exogenously-applied 5-aminolevulinic acid and hydrogen sulfide enhanced the photosynthetic and antioxidant capacity of cold-sensitive plants.

Additional insights into the molecular mechanisms of low temperature tolerance in plants were provided by the observation that the *Vitis amuriensis* gene *VaAPL1*, encoding the APL1 subunit of ADP-glucose pyrophosphorylase, a rate-limiting enzyme in starch biosynthesis, promotes low temperature tolerance when overexpressed in *Arabidopsis* and tomato (*Solanum lycopersicum*) (Liang et al.). ROS content was lower in VaAPL1-overexpressing lines compared to the wild-type plants after cold exposure, while sucrose and glucose accumulated. Moreover, some genes in the hormone- and Ca^2+^-signaling pathways were also up-regulated in the overexpressing plants. These results therefore suggest a role for starch mobilization in the maintenance of cell osmotic potential and energy supply required to withstand cold stress.

Lurie reviewed published literature on proteomic and metabolomics related to chilling injury in peach and nectarine (*Prunus persica*) fruit, which are very prone to develop visible symptoms when stored at temperatures within the so-called “killing zone” (roughly 2-8 °C) (Lurie and Crisosto, 2005). Chilling injury damage in these produce encompasses many internal and external disorders which negatively affect the commercial quality of fruit, and limit their storage and shelf life potential. This survey indicated that chilling exposure decreases the expression of proteins involved in ethylene biosynthesis, hence affecting ripening-related events such as cell wall changes and the emission of aroma volatiles. Chilling-injured fruit suffer from imbalanced energy production and produce more ‘stress-proteins’. Sugars and sugar derivatives, as well as polyamines as membrane stabilizers, play a role in protection against chilling injury. In contrast, no conclusive results have been reported so far on a possible role therein of amino acids or some lipid species.

Chilling injury in tomato fruit manifests as physiological disorders and decay after storage at low temperatures. In an attempt to investigate the responses of tomato fruit to postharvest cold stress, David et al. performed a comparative study of a number of recombinant inbred lines (RIL) derived from a cross between cultivated tomato (chilling-sensitive) and a chilling-tolerant inbred accession of *S. pimpinellifolium*, a wild tomato species. Screening of cold responses in fruit from 148 RILs showed large variation in chilling tolerance. Two RIL groups with contrasting differences in cold tolerance were selected for further investigation. Photosynthetic parameters such as Fv/Fm and performance index were found to be efficient markers for the early identification of chilling responses. Chilling-tolerant lines had higher antioxidant activity, ascorbic acid content, and gene expression levels of C-repeat/DREB binding factors, which are central for cold acclimation in plants (Shi et al., 2018).

## Treatments for the alleviation of cold stress

Since increased ROS contents are related to chilling injury, exogenous application of antioxidant compounds might help to improve cold tolerance. Liu et al. applied a hydrogen-rich water (HRW) treatment to seedlings of *Tetrastigma hemsleyanum*, a cold-sensitive species within the *Vitaceae* family. Exogenous HRW might help plants release endogenous H_2_, which in turn could contribute to improving stress resistance (Cao et al., 2017). Integrative metabolic and transcriptomic analyses demonstrated that HRW application could alleviate stress damage by decreasing stomatal density and increasing photosynthetic efficiency. Results indicated a major role for the phenylpropanoid biosynthesis pathway in cold stress responses, the alteration in which was mitigated by exogenous HRW, and a strong relationship between total antioxidant capacity and transpiration rate was observed.

Postharvest handling of banana (*Musa* spp., AAA group, Cavendish type), a climacteric fruit species, is severely limited because of proneness to chilling injury. Yet, if fruit are stored at their chilling threshold temperature (CTT, the lowest storage temperature avoiding chilling injury), ripening will proceed. Chilling stress-induced ethylene could be involved in the development of chilling symptoms. In order to explore this possibility, ethylene action was inhibited by means of 1-methylcyclopropene (1-MCP) application (Chang et al.). Results demonstrated that 1-MCP application allowed mature green bananas to be stored at their CTT, while the onset of ripening was delayed when compared to the untreated controls. Vascular browning and light-adapted quantum yield of photosystem II were shown to be good indicators of early chilling stress in these fruit.

## Conclusions

The papers in this Research Topic used physiological, biochemical and molecular approaches to explore cold stress tolerance in plants. Improved resistance to chilling stress was often associated with enhanced antioxidant activity and osmolyte content. Photosynthetic parameters may be used as early indicators of chilling injury, while antioxidant treatments may help with symptom alleviation.

## Author contributions

All authors listed contributed directly to manuscript preparation, and revised and approved it before submission.

## Funding

Current work at IL’s lab is funded by grant 2017 SGR 1108 (Generalitat de Catalunya, Catalonia, Spain). DB acknowledges funding from the US-Israeli Binational Agricultural Research Development Fund and the AES Hatch Project CA-D-PLS-2404-H. Work at MD’s lab is funded by National Research Council and National Agency for the Promotion of Scientific and Technological Activities from Argentina.

## Conflict of interest

The authors declare that the research was conducted in the absence of any commercial or financial relationships that could be construed as a potential conflict of interest.

## Publisher’s note

All claims expressed in this article are solely those of the authors and do not necessarily represent those of their affiliated organizations, or those of the publisher, the editors and the reviewers. Any product that may be evaluated in this article, or claim that may be made by its manufacturer, is not guaranteed or endorsed by the publisher.
